# Group-Level Ranking-Based Hubness Analysis of Human Brain Connectome Reveals Significant Interhemispheric Asymmetry and Intraparcel Heterogeneities

**DOI:** 10.3389/fnins.2021.782995

**Published:** 2021-12-21

**Authors:** Sahin Hanalioglu, Siyar Bahadir, Ilkay Isikay, Pinar Celtikci, Emrah Celtikci, Fang-Cheng Yeh, Kader Karli Oguz, Taghi Khaniyev

**Affiliations:** ^1^Department of Neurosurgery, Hacettepe University Faculty of Medicine, Ankara, Turkey; ^2^Department of Radiology, Ankara City Hospital, Ankara, Turkey; ^3^Department of Neurosurgery, Gazi University Faculty of Medicine, Ankara, Turkey; ^4^Department of Neurological Surgery, University of Pittsburgh, Pittsburgh, PA, United States; ^5^Department of Radiology, Hacettepe University Faculty of Medicine, Ankara, Turkey; ^6^National Magnetic Resonance Research Center (UMRAM), Bilkent University, Ankara, Turkey; ^7^Department of Industrial Engineering, Faculty of Engineering, Bilkent University, Ankara, Turkey; ^8^Sloan School of Management, Massachusetts Institute of Technology, Cambridge, MA, United States

**Keywords:** graph theory, connectome, network, population, hub, parcellation, hubness, hemispheric asymmetry

## Abstract

**Objective:** Graph theory applications are commonly used in connectomics research to better understand connectivity architecture and characterize its role in cognition, behavior and disease conditions. One of the numerous open questions in the field is how to represent inter-individual differences with graph theoretical methods to make inferences for the population. Here, we proposed and tested a simple intuitive method that is based on finding the correlation between the rank-ordering of nodes within each connectome with respect to a given metric to quantify the differences/similarities between different connectomes.

**Methods:** We used the diffusion imaging data of the entire HCP-1065 dataset of the Human Connectome Project (HCP) (*n* = 1,065 subjects). A customized cortical subparcellation of HCP-MMP atlas (360 parcels) (yielding a total of 1,598 ROIs) was used to generate connectivity matrices. Six graph measures including degree, strength, coreness, betweenness, closeness, and an overall “hubness” measure combining all five were studied. Group-level ranking-based aggregation method (“measure-then-aggregate”) was used to investigate network properties on population level.

**Results:** Measure-then-aggregate technique was shown to represent population better than commonly used aggregate-then-measure technique (overall r_s_: 0.7 vs 0.5). Hubness measure was shown to highly correlate with all five graph measures (r_s_: 0.88–0.99). Minimum sample size required for optimal representation of population was found to be 50 to 100 subjects. Network analysis revealed a widely distributed set of cortical hubs on both hemispheres. Although highly-connected hub clusters had similar distribution between two hemispheres, average ranking values of homologous parcels of two hemispheres were significantly different in 71% of all cortical parcels on group-level.

**Conclusion:** In this study, we provided experimental evidence for the robustness, limits and applicability of a novel group-level ranking-based hubness analysis technique. Graph-based analysis of large HCP dataset using this new technique revealed striking hemispheric asymmetry and intraparcel heterogeneities in the structural connectivity of the human brain.

## Introduction

Human brain operates as a confined, compact and complex dynamic network. Understanding the structure and dynamics of this global network is essential to understand how human brain has evolved to perform highly sophisticated functions, as well as to reveal how brain pathologies lead to neuropsychiatric manifestations by disrupting that network, which, in turn, could pave the way for novel interventions to modulate the pathological subnetworks to provide treatments for these conditions (van den Heuvel and Sporns, 2013; Herbet and Duffau, 2020).

Advancements in basic neuroscience and imaging techniques have provided us with unprecedented opportunities to study the brain. Large imaging data sets accessible to all researchers, new data analytical tools and research collaborations accelerated discovery in the field of human network neuroscience (Van Essen et al., 2012).

It has been shown that the complex structure of the brain can be represented mathematically as a graph (Hagmann et al., 2008). Graph theoretical methods have gained attention for examining cortical and subcortical connections, networks, subnetworks and transient “meta networks.” In their seminal article, Hagmann et al. (2008) have shown that it is possible to determine a structural core for the brain network and they illustrated how it is possible to model complex brain networks with graphs, understand its network features, find nodes exhibiting hubness property, uncover connection pathways and even alternative pathways. Later research has also shown that it is possible to understand how the energy economy is optimized within this network (Bullmore and Sporns, 2012). Other studies have also shown that different brain areas act as hubs (van den Heuvel and Sporns, 2013; Khaniyev et al., 2020).

There is now an emerging concept of “minimal common brain,” consisting of most fundamental tracts and connections (Herbet and Duffau, 2020). Early studies (Hagmann et al., 2008) were successful in identifying hub regions and other important regions which have central roles in signal transmission between different parts of the brain. These studies, however, were suffering from low sample size and high variability. To the best of our knowledge, the findings of Hagmann et al. (2008) have not been systematically reproduced on a different, larger dataset that can represent the population better. Adding to that, it is still an open question how best to represent inter-individual differences with graph theoretical methods.

Although graph theory has been successfully utilized in many network neuroscience studies including healthy subjects as well as those with neurological disorders, keeping the aforementioned shortcomings in mind, we aimed to confirm and/or challenge some of the previous findings in the field with a large dataset and a consistent methodology. At the center of our investigation lies the identification of hub regions in the connectomes of 1,065 healthy subjects included in the Human Connectome Project (HCP) dataset. To quantify the differences/similarities between different connectomes, we proposed a simple intuitive method that is based on finding the correlation between the rank-ordering of nodes within each connectome with respect to a given metric. Using this simple approach, we measured and compared the effectiveness of different aggregation methods in representing the population. Finally, we made use of the proposed approach to measure and reveal the level of asymmetry between hemispheres and the heterogeneities within some brain areas, thereby challenging some of the existing assumptions regarding the interhemispheric symmetry and intraparcel homogeneity with respect to hubness property.

## Materials and Methods

### DTI Data Acquisition and Tractography

#### MRI Dataset Specifications

We used HCP-1065 dataset for human tractography. The minimally-preprocessed data (Glasser et al., 2013) from the Human Connectome Project (Q1–Q4 release, 2015) were acquired by Washington University in Saint Louis and University of Minnesota (Van Essen et al., 2012). The diffusion MRI scans were conducted on a Siemens 3T Skyra scanner using a 2D spin-echo single-shot multiband EPI sequence with a multi-band factor of three and monopolar gradient pulse. The sequence applied a TR of 5,500 ms and a TE of 89.50 ms with 1.25 mm isotropic spatial resolution. A Multi shell diffusion MRI sampling technique was used (Caruyer et al., 2013; Sotiropoulos et al., 2013) and the b-values were 1,000, 2,000, and 3,000 s/mm^2^ respective to the shells. The total number of diffusion sampling directions was 90, 90, and 90 for each of the shells in addition to six b0 images. Original data were then pre-processed in accordance with the minimal preprocessing pipelines of the HCP to achieve spatial artifact/distortion removal, surface generation, cross-modal registration, and alignment to the MNI standard space (Glasser et al., 2013). The preprocessed data were corrected for eddy current and susceptibility artifacts.

#### Pre-processing of Diffusion Imaging and Tractography

DSI studio was used for pre-processing and reconstruction of diffusion imaging and tractography. The in-plane resolution of images were 1.25 mm. The slice thickness were 1.25 mm. The b-table was checked by DSI Studio through an automatic quality control routine to ensure its accuracy (Schilling et al., 2019). The diffusion data were reconstructed in the MNI space using q-space diffeomorphic reconstruction (Yeh and Tseng, 2011) to obtain the spin distribution function (Yeh et al., 2010). A diffusion sampling length ratio of 1.25 was used. The output resolution were 1 mm isotropic. The restricted diffusion was quantified using restricted diffusion imaging (Yeh et al., 2017).

#### Fiber Tractography

Deterministic fiber tracking method provided by DSI Studio was used for fiber tractography (Yeh et al., 2013). Tracking parameters were set as follows; Otsu threshold was set to 0.6, fractional anisotropy threshold determines threshold for fiber termination and is used as a mask to filter out background voxels, it was determined automatically by Otsu threshold and set to 0.6, turning angle was assigned to 0 to do a random selection between 15 and 90 degrees. This threshold also acts as a criterion for fiber termination, above which DSI studio terminates the fiber if two consecutive moving directions have crossing angle. Step size defines moving distance in each tracking interval, the unit is in millimeters and was set to 0 in order to make a random selection between 0.5 and 1.5 voxel distance. Smoothing parameter determines the moving direction with regard to previous propagation vector. Smoothing was set to zero so the propagation direction was independent of previous incoming direction. Minimum and maximum lengths of fibers were set to 30 and 300 mm, respectively. Tracts were created from 1,000,000 seeds for every subject, and an average of 500,000 tracks were created for each subject. For a handful of subjects (*n* = 5), we ran the tractography with different numbers of seeds (up to 20,000,000) and observed very similar results with respect to the hubness ranking beyond 1,000,000 seeds.

#### Normalization to Montreal Neurological Institute Space

The Montreal Neurological Institute (MNI)–International Consortium for Brain Mapping ICBM 152 space has been created in response to the need for a system that provides an organized and manageable means for collecting, comparing and rapidly searching for data, hypothesis generation and testing novel theories in brain sciences, respecting the brain anatomy (Maziotta et al., 2001). It provides a template brain to which target brain can be warped into, by means of linear or non-linear computational methods. In our study, we used q-space diffeomorphic reconstruction method to register our target brains to ICBM-152 space, as proposed previously (Yeh and Tseng, 2011) in order to overcome known difficulties associated with diffusion tensor imaging such as crossing fibers problem (Alexander et al., 2002) and partial volume problem (Alexander et al., 2001; Fritzsche et al., 2010).

#### Atlas Selection

**FIGURE 1:**
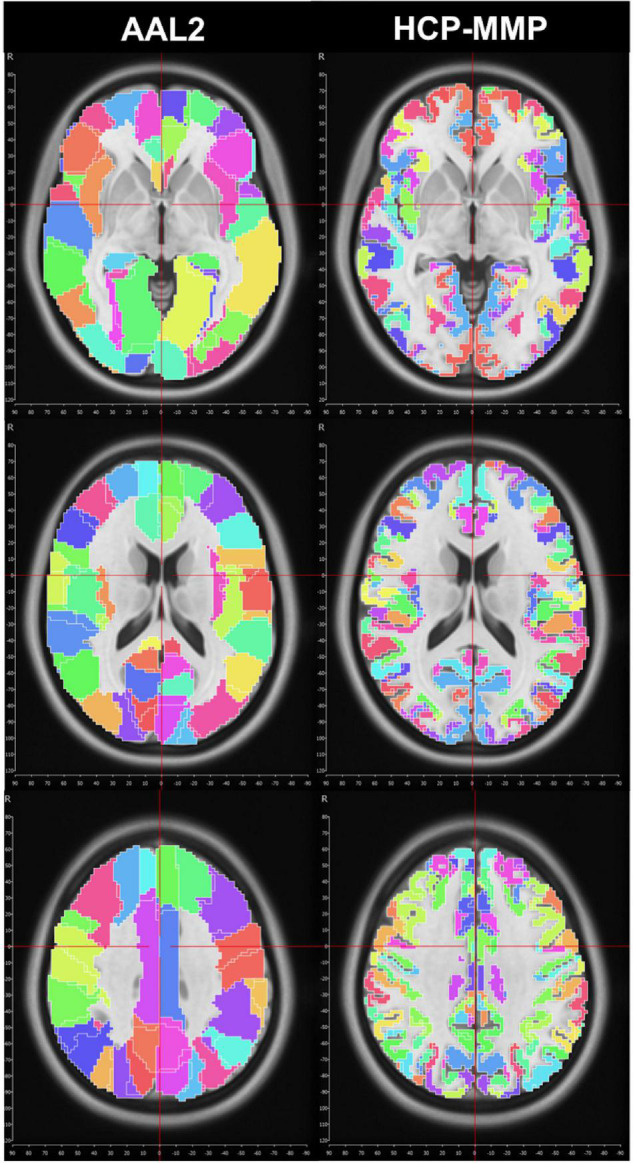
Midline crossing in two different parcellation atlas. While midsagittal plane according to MNI space violated left medial hemispheric cortex in the AAL2 atlas, this effect was minimal, if any, in HCP-MMP atlas.

We first used the AAL2 atlas (Tzourio-Mazoyer et al., 2002) to identify cortical regions (parcels) and subsequently create subparcellations for graph analysis. However, the network analysis results showed a distinct asymmetry between hemispheres that pointed to a technical artifact involving midline standardization. Troubleshooting process revealed that standard MNI space coordinates of the AAL2 atlas have a slight midline shift in the left to right direction which causes some medial cortical areas of the left hemisphere to be included in the right hemisphere erroneously (Figure 1). Thus, we selected a more detailed and newer parcellation atlas (HCP-MMP) derived based on the HCP dataset itself (Glasser et al., 2016). Although a similar trend was also observed for the HCP-MMP atlas, the magnitude of the shift was significantly smaller in HCP-MMP compared to that in AAL2 atlas (Figure 1).

#### Sub-Parcellation

**FIGURE 2:**
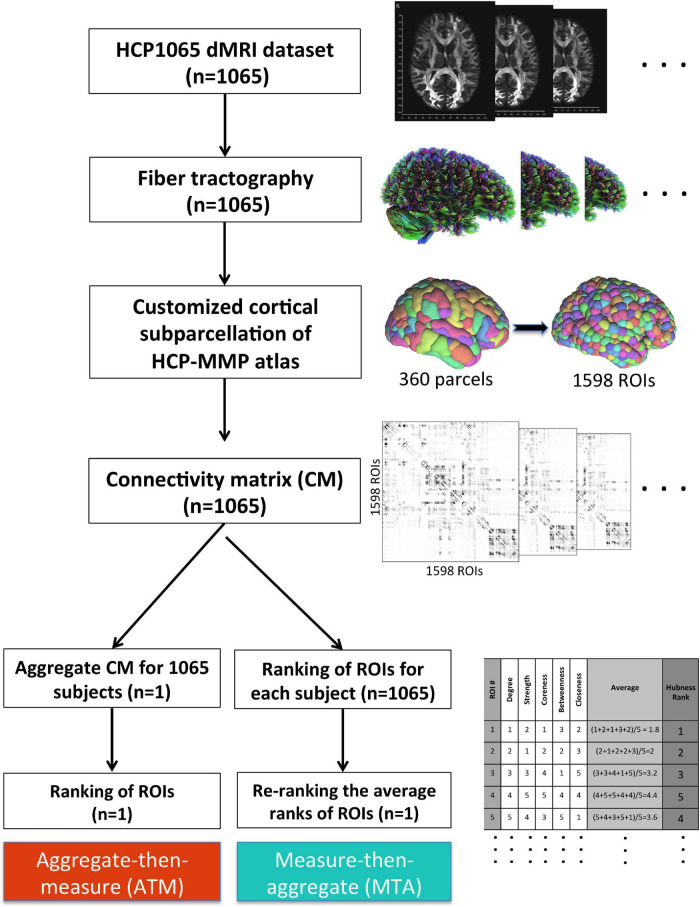
Methodological workflow of the study. We acquired the minimally-preprocessed diffusion MRI data from HCP1065 dataset (*n* = 1,065 subjects). Then, we performed whole-brain tractography for each subject using DSI Studio software. We adopted HCP-MMP atlas (360 parcels) for cortical parcellation. However, in order to minimize the effect of parcel size, we created subparcels (ROIs) within the original parcel boundaries to obtain ROIs of similar sizes (*n* = 1,598 ROIs). Then, the 1,598 × 1,598 connectivity matrix (CM) for each subject was generated. After that, two different methods for group-level analysis (ATM and MTA) were employed in parallel. Whereas more conventional ATM (aggregate-then-measure) technique generates an average CM for the population first (“aggregate”) and ranks ROIs with respect to given graph metric afterward (“measure”), MTA (measure-then-aggregate) technique creates separate connectivity matrices, ranks the ROIs with respect to the given graph metric for each subject first (“measure”), and then averages them across subjects (“aggregate”) to have a representation of the large population. Ranking approach is also illustrated in table located on the bottom right of the figure.

HCP-MMP atlas consists of 360 distinct parcels (180 in each hemisphere) with vastly different sizes. The largest parcel (7,103 mm^3^) in HCP-MMP atlas is approximately 30 times bigger in volume than the smallest parcel (236 mm^3^). To alleviate this heterogeneity in sizes, we further split each parcel into smaller spatially contiguous regions of interests (ROIs). We clustered voxels within each parcel using a simple k-means clustering algorithm that minimizes the average distance between the voxels within the same cluster. With this approach, parcels (360 in total) were divided into smaller subparcels or ROIs (1,598 in total), which were more homogenous in size (Figure 2). Homogenization of sizes *via* sub-parcellation made a fair node-by-node comparison possible with respect to various network measures in the subsequent analyses. However, since their homology between two hemispheres is not dictated by the algorithm to produce the sub-parcellation within the same parcel of each hemisphere, we used parcels instead of ROIs to assess interhemispheric differences. When switching from ROIs to parcels, we took into account the median ranking of subparcels (ROIs) within a given parcel.

It is worth mentioning that the total number of ROIs (1,598) was not deliberately chosen. As we applied k-means clustering to each parcel’s voxels separately, we needed to predefine the number of clusters (k) for the algorithm to run. Considering the sizes of the smallest parcels (<400 voxels), we decided not to divide them up further. For the rest of the parcels, the number k was determined by dividing the total number of voxels in that parcel by 400 and taking the integer quotient. e.g., if the total number of voxels in a parcel was 3,000, then the *k* = (3,000/400) = 7 was chosen.

#### Construction of Structural Connectivity Networks

To construct the structural connectivity networks, first each ROI generated in the sub-parcellation step was represented with a node. Then, each pair of nodes whose corresponding ROIs were the two endpoints of at least one fiber connection in the tractography step was connected with an edge. To be able to provide a direct comparison with the results from Hagmann et al. (2008), we adopted the following formula from their study to calculate the weight, w_ij_, of an edge between the nodes i and j:


$${\text{w}}_{\text{ij}}={\text{n}}_{\text{ij}}*\left(1/{\text{L}}_{\text{ij}}\right)*\left(2/\left({\text{V}}_{\text{i}}+{\text{V}}_{\text{j}}\right)\right)$$

where n_ij_ is the number of fibers whose two endpoints are nodes i and j, L_ij_ is the mean length of those fibers, and V_i_ (V_j_) are the total number of voxels in the ROIs corresponding to the nodes i and j, respectively. From this weighted network, we also obtain a binary unweighted network by connecting all pairs of nodes which have an edge between them in the weighted graph with a single edge of unit weight. In the above formula, the correction term 1/L_ij_ is used because the tractography algorithm used by DSI Studio introduces a bias which causes a higher fraction of false positive tracts for the longer fibers. And the other normalization term [2/(V_i_+V_j_)] is used to correct the slight differences in the sizes of the ROIs obtained by sub-parcellation.

*Comparative Network analysis:* In the literature, there are two levels of analysis comparing brain connectivity networks: (i) global (network-level) comparison, (ii) local (node-level) comparison (Meskaldji et al., 2013). On global level analysis, for each graph-theoretical measure studied (such as global efficiency, clustering coefficient, small-worldedness), a single value is computed for a given network. Although this approach may give us a rough idea about how similar two distinct networks are, the drawback is that two very different networks may have exactly the same value with respect to the measure used, which may falsely be interpreted as the two networks being identical. To alleviate this problem, a local (node-level) analysis may be preferred, where for each graph-theoretical measure studied (such as degree, strength, coreness, efficiency, centrality), a single value is computed for each node. In order to quantify the similarity/dissimilarity of two networks *via* local analysis, one needs to conduct multiple pairwise node-by-node comparisons and aggregate the results from individual comparisons in a mathematically rigorous way (Meskaldji et al., 2013). We propose a simple ranking-based approach to quantify the similarity of any two networks with the same number of nodes. First, we separately rank the nodes in each network using a given node-level graph-theoretical measure (including well-known measures such as degree, coreness, centrality as well as customized measures such as hubness). For example, if the degree values for five ROIs are (50, 20, 30, 40, 10), then the ranking vector is (1, 4, 3, 2, 5) where ROI#1 is the top-ranking ROI with respect to the degree measure. To compare two ranking vectors obtained in this way, we use the Spearman correlation coefficient:


$$\rho =1-\frac{6\,\sum d_{i}^{2}}{n\,\left(n^{2}-1\right)}$$

where

ρ : Spearman’s rank correlation coefficient (r_s_).

*d*_*i*_:difference between the two ranks of each observation.

*n*: number of observations.

Spearman correlation of two ranking vectors can take values between −1 and 1 and the closer it is to 1, the more similar the two networks are in terms of the considered graph-theoretical measure. This way, similarity of two networks is quantified in a standardized way by a single value derived from multiple node-level values.

#### Hubness Measure

One of the important problems of the network neuroscience domain is to identify the regions in the human brain that play a hub role and act as an intermediary to transmit signals between different regions. Hagmann et al. (2008), in their seminal paper, rigorously investigated the hubness property of different regions in the human brain cortex using graph-theoretical measures. They argue that a hub region must be highly connected and topologically centrally located. To that end, they use the following graph-theoretical measures of a node as a proxy to determine the extent the node exhibits hubness property:

• *Degree* of a node is the number of edges that are incident to it.
• *Strength* of a node is the total weight of the edges that are incident to it.
• *Coreness* is a measure to identify tightly interlinked nodes within a network. A k-core is a maximal subset of nodes, all of which are connected to at least k other nodes in the subset. The coreness of a node is k if it belongs to the k-core but not to the (k+1)-core.
• *Betweenness* of a node is the fraction of the unweighted shortest paths among all pairs of nodes that pass through the node.
• *Closeness* of a node is the reciprocal of the sum of the unweighted shortest path distances between the node and all other nodes.

Hagmann et al. (2008) compute the hubness rank of each node in a connectome network by an elaborate method combining the ranks with respect to the aforementioned measures. In this study we opted for using a simpler approach of averaging the ranks with respect to each measure which provided very similar hubness ranks to those obtained by Hagmann et al. (2008)’s method.

#### Group-Level Aggregation

Traditional approach to calculating the group-level rankings of nodes with respect to the graph-theoretical measures is to first obtain an aggregate connectome for the group by averaging the connectivity matrices of all subjects within the group and then calculating the values of network measures for each node in the aggregated connectome (Figure 2). In this study, we call this approach “Aggregate-then-Measure” (ATM). An alternative approach is to first calculate the values of the network measures for each individual connectome and then aggregate them by averaging the values for each node across individuals within the group. We call this approach “Measure-then-Aggregate” (MTA). One of the contributions of this study is to illustrate that the latter approach generates aggregate rankings with a significantly higher representative power compared to the former.

## Results

We compiled our experimental findings into two main sections each with a different focus.

### Section I: Methodological Insights for Connectome Network Analysis at Population Level

#### Result 1: An Alternative Aggregation Method for Robust Representation of Population Connectomics

In studies involving connectivity data from multiple subjects, an important step is to determine how to aggregate the data from different subjects in such a way that the resulting group-level aggregated data better represents the population with respect to a given metric (e.g., degree, strength, etc.). To this end, we tested the representation power of two different approaches:

• **Aggregate-then-Measure:** First aggregate the individual connectomes into a single group connectome, then rank the nodes/ROIs with respect to the given metric to get the group-level ranking (Ranking_ATM).
• **Measure-then-Aggregate:** First rank the ROIs with respect to the given metric FOR EACH subject, independently, then average the ranking of each ROI across subjects to get the group-level-ranking (Ranking_MTA).

**FIGURE 3:**
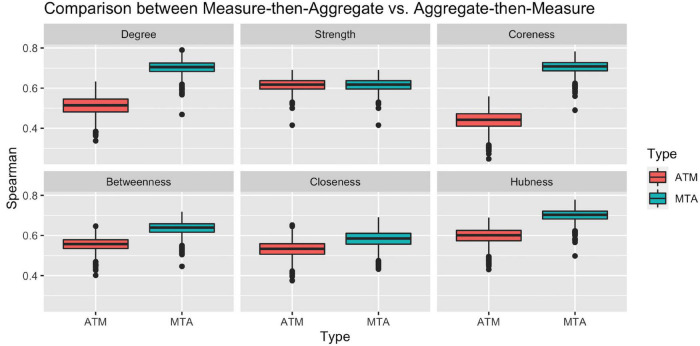
Comparison between two population-averaging techniques. Measure-then-aggregate (MTA) technique fare significantly better than aggregate-then-measure (ATM) technique to represent network properties of population.

To check the representation power of the above approaches, we calculated the Spearman ranking correlation of Ranking_ATM and Ranking_MTA with each individual ranking in the population. Figure 3 clearly illustrates that with respect to all network measures (except for strength, where the two approach ties), the ranking obtained by Measure-then-Aggregate approach has significantly higher correlation, on average, with the individual subjects compared to Aggregate-then-Measure approach. For example, with respect to degree, the group-level ranking obtained by Measure-then-Aggregate has, on average, approximately 0.7 correlation with the individual rankings of the subjects; whereas the average correlation of the group-level ranking obtained by Aggregate-then-Measure approach is only about 0.5.

We conclude from Figure 3 that when the end goal is to make inference about a population by using the connectome data from a number of subjects (not necessarily requiring to obtain a group-level connectome explicitly), it is more appropriate to first calculate the network measure of interest from each subject’s connectome and then take an average of that measure instead of first finding the aggregate connectome and then calculating the measure of interest for the aggregate connectome. The Measure-then-Aggregate approach produces significantly more representative rankings for the population compared to the Aggregate-then-Measure approach which is the method many studies use in the literature (Simpson et al., 2012; Gleichgerrcht et al., 2015; Wang et al., 2015; Yeh et al., 2018; Aggarwal and Gupta, 2019; Hallquist and Hillary, 2019; Domhof et al., 2021).

#### Result 2: An Overall Hubness Measure Combining Different Graph Measures

**FIGURE 4:**
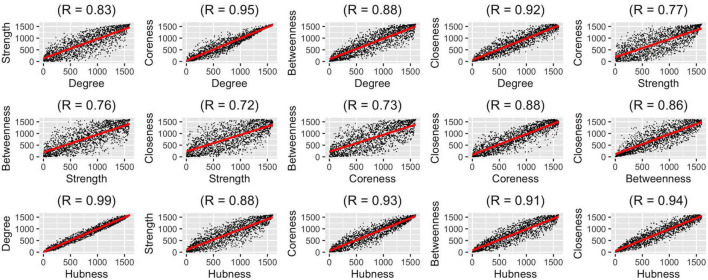
Pair-wise correlation analysis of six graph measures using group-level ranking-based analysis technique.

After determining which aggregation method should be used for representing the population best, we wanted to compare how different graph measures correlate with each other on the group-level. We used five graph measures and an overall hubness measure which we derived by averaging these five graph measures. We conducted pairwise correlation analysis of these six graph measures (Figure 4). In these analyses, correlation values (r_s_) were between 0.72 and 0.99, highest correlations were between degree-hubness: 0.99, closeness-hubness 0.94, coreness-hubness 0.93 pairs. When the overall hubness measure is excluded, the first three correlations are coreness-degree: 0.95, closeness-degree: 0.92 ve closeness-coreness 0.88.

We observed that most of the graph measures are very highly correlated with each other. Considering the insight that when using highly-correlated variables to infer a dependent variable (hubness, in our case), one needs to pay close attention to how the variables are weighted, we pose the question whether it makes sense to assume all graph measures are equally weighted in determining hubness. To properly answer this question, however, a more in-depth analysis is needed which is out of the scope of this paper.

#### Result 3: Minimum Number of Subjects to Study Connectomics on Population Level

To illustrate the effect of sample size in the representation power of a randomly drawn sample, we conducted the following computational analysis. For each n_s_ = {1, 2, 5, 10, 20, 50, 100, 200, 500, 1,000}, we first draw a random sample of size n_s_ from the population of *n* = 1,065 subjects. Then, we calculate the sample-averaged hubness ranking and find its Spearman correlation with the population-averaged hubness ranking. Assuming the population-averaged ranking is our ground truth, we would like to achieve as high correlation as possible.

**FIGURE 5:**
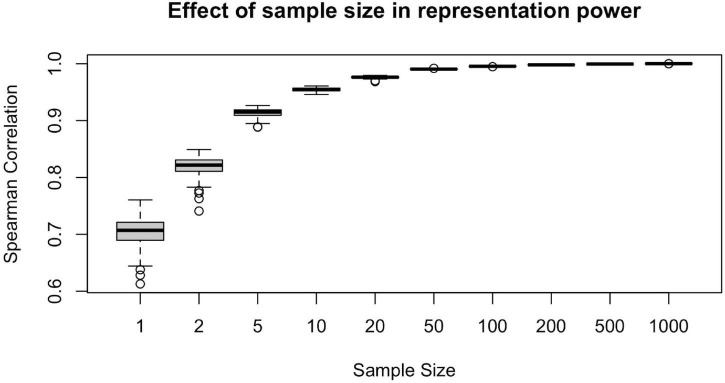
Effect of sample size on the representative power for population. Representative power increases with sample size up to 50 subjects, however, beyond *n* = 50, little gain is observed since the plateu is reached at > 99% accuracy.

We calculated the Spearman correlation of the population-averaged hubness ranking with the sample-averaged hubness ranking of 100 different samples for each n_s_ = {1, 2, 5, 10, 20, 50, 100, 200, 500, 1,000} value and plot resulting correlation coefficients against the sample size (Figure 5). We observe that at around n_s_ = 50, the sample-averaged hubness ranking gets sufficiently close to the population-averaged hubness ranking (mean r_s_ = 0.99, range: 0.988–0.992). Increasing the sample size beyond n_s_ = 50 does not seem to add much value in terms of the accuracy of the hubness ranking (e.g., for n_s_ = 100, mean r_s_ = 0.995, range: 0.994–0.996). However, below n_s_ = 50, the quality of the sample-averaged hubness ranking seems to decrease significantly and vary depending on the specific sample chosen. For example, for n_s_ = 5, the Spearman correlation of sample-averaged hubness ranking with the population-averaged hubness ranking is 0.896 for one sample and 0.926 for another. Based on these findings, 50 to 100 randomly selected subjects are required to have a sufficiently high representation power.

### Section II: The Results of Population-Based Network Analysis of Brain Hubs

#### Result 4: Mapping Cortical Hubs Identified From Population-Based Network Analysis

**FIGURE 6:**
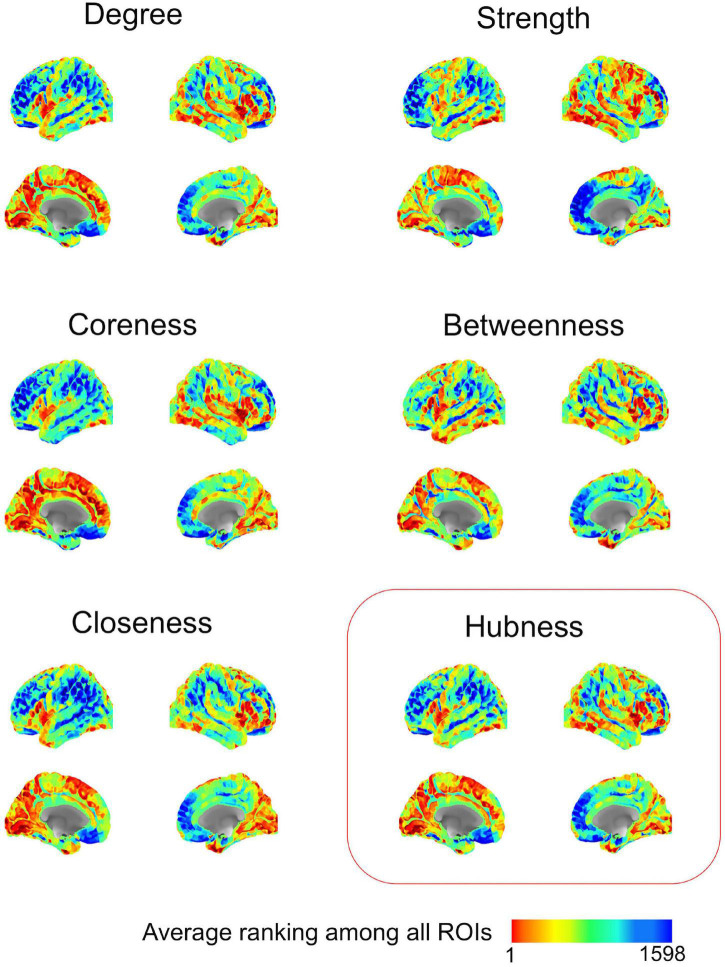
Ranking heatmaps of cerebral cortex for different graph measures on population-level. High rank regions (with lower ranking values) are depicted in red color; low rank regions (with higher ranking values) are depicted in blue color.

**FIGURE 7:**
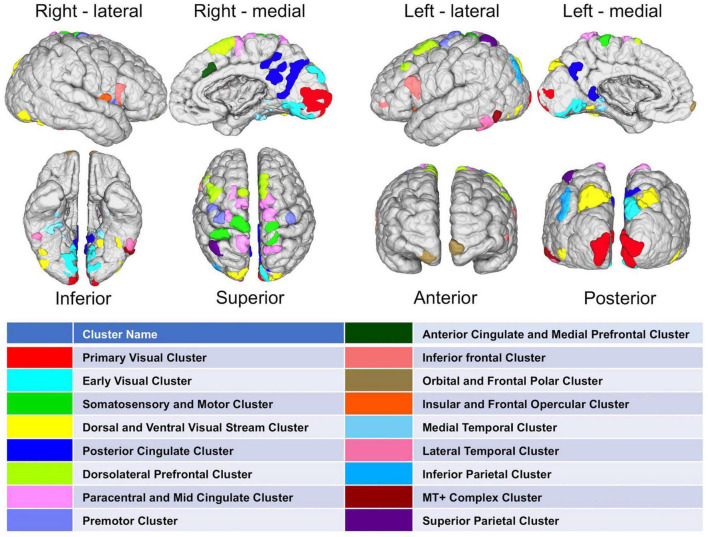
Distribution of highly-connected hub clusters. Cortical regions ranking on top 20% in all five graph measures on population-level are depicted. Subparcels (ROIs) regarded as highly-connected hubs are clustered using corresponding HCP-MMP cortices.

After methodological optimization, we produced heatmaps of average rankings of the cortical ROIs for each graph measure (Figure 6). Although heatmaps for individual graph measures slightly differ from one another, there is a considerable overlap between them as expected from their intercorrelation. We used the overall “hubness” graph measure to characterize highly consistent cortical hubs on population level. As visualized in the Figure 6, almost entire medial surface of the left hemisphere, medial temporo-occipital region of the right hemisphere, bilateral superior frontal gyri, ventral premotor areas, pars opercularis of inferior frontal gyri, right posterior temporal regions appear to be hub regions. We also identified the ROIs in the top 20% of ranking for all five graph measures. A total of 131 ROIs with similar numbers between two hemispheres (65 ROIs in the Right, 66 ROIs in the Left) were found. Clustering of those ROIs revealed critical cortices with hubness property (Figure 7).

#### Result 5: Hemispheric Asymmetry of Homologous Brain Regions in Network Measures

**FIGURE 8:**
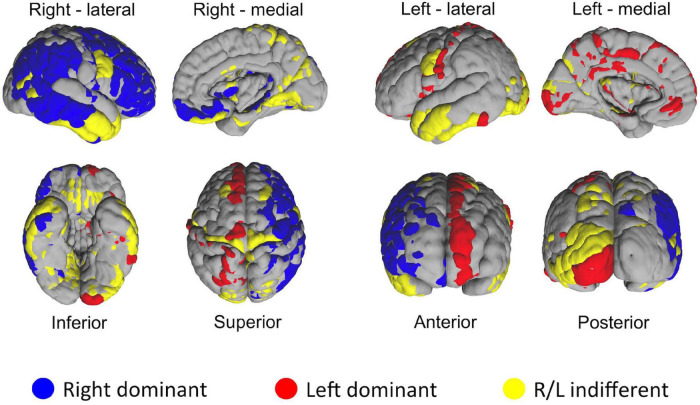
Distribution of HCP-MMP parcels in two hemispheres based on the significance of average ranking differences. If a given homologous parcel is ranked significantly different between two hemispheres, it is depicted in either blue (R dominant/high rank) or red (L dominant/high rank). Yellow color indicates no significant difference in ranking between homologous parcels of two hemispheres.

One of the distinct features of the heat maps was hemispheric asymmetry. While certain homologous regions (parcels) ranked similarly across two hemispheres, others did not. We next investigated how similar was the ranking of homologous regions between two hemispheres by using correlation analysis. Overall, correlation coefficient between two hemispheres was 0.64. Next, we asked how the rankings of individual homologous parcels differ between two hemispheres. In the first set of experiments we defined a 5% margin as an acceptable difference between rankings on population level. This analysis revealed that, out of 180 homologous parcels, 52 were not different between hemispheres (yellow), whereas 75 has right (blue), 53 had left (red) hemispheric dominance (i.e., had significantly (*p* < 0.01) higher ranks in one hemisphere than another) (Figure 8). In the second experiment, we checked whether the median hubness ranking of a parcel has an impact on the level of asymmetry between two hemispheres, but could not find any significant correlation (r_s_ = 0.03).

#### Result 6: High Level of Heterogeneity Within Cortical Parcels

**FIGURE 9:**
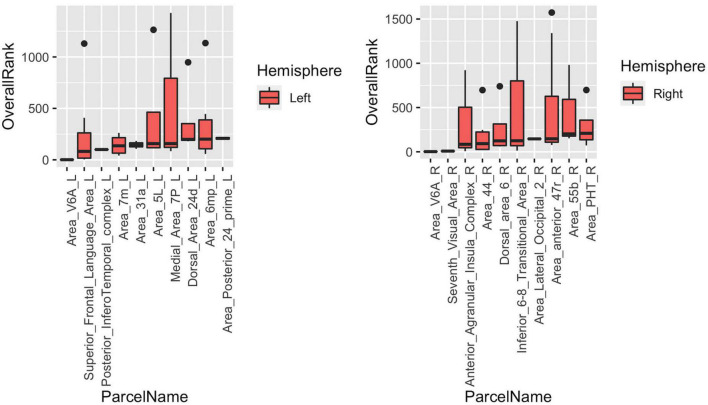
Box-blots representing subparcel rankings within top 10 parcels for hubness measure in each hemisphere. Each boxplot represents the variance in the population-level hubness ranking (obtained by using MTA approach) of ROIs within the given parcel (shown in x axis). Therefore, the variance of population-level hubness ranking within parcels consisting of a single ROI (such as Area_V6A_L, Posterior_Infero Temporal_complex_L, etc.) is zero. Distinct intraparcel heterogeneity is clearly visible in other parcels.

The last part of the study focused on characterizing heterogeneity within individual cortical parcels. Here, we showed that even with a very detailed and well-studied cortical parcellation atlas like HCP-MMP, there is a high level of heterogeneity within parcels when graph features are concerned. In the Figure 9, top 10 parcels of each hemisphere are shown with box-plots representing individual rankings of ROIs within each parcel. Each boxplot represents the variance in the population-level hubness ranking (obtained by using MTA approach) of ROIs within the given parcel (shown in x axis). As clearly visible from box-plots, some parcels have very high level of intraparcel heterogeneity (for example Medial_Area_7P_L, Area_44_L, Parahippocampal_Area_1_L, Second_Visual_Area_L, Anterior_Agranular_Insula_Complex_R, Inferior_6-8_Transitional_Area_R, Area_anterior _47r_R) with ranks within the same parcel ranging from top 10% to bottom 10% (see Medial_Area_7P_L).

## Discussion

The Human Connectome Project has transformed neuroscience research and provided researchers with an enormous support for the acquisition, analysis, visualization, mining and sharing of connectome-related data (Marcus et al., 2011; Van Essen et al., 2012). Different groups across the globe can now use this open-access big data to generate new information on human brain connectivity. In addition, new methodologic approaches provide novel insights into the analysis and interpretation of this data. In this study, we have introduced and tested a new mathematical (or computational) approach to analyze network characteristics of human brain connectome on group or population level with a different perspective. In the first set of findings, we provided experimental evidence for the robustness, limits and applicability of this new averaging technique based on ranking of individuals in certain graph measures. In the second section, we presented the novel findings and insights on the graph-based analysis of large HCP dataset using this new technique. This analysis revealed striking hemispheric asymmetries and intraparcel heterogeneities in the structural connectivity of the human brain. We will discuss in the following sections each main finding of this study.

First, we compared the two principal averaging methods for graph-based connectome analysis of multiple subjects to identify the optimal approach for more accurate representation of the population. A more traditional approach for group-level or population-level brain networks is to build a single composite or aggregate graph by simply averaging graph metrics of each individual, and then investigating network measures based on this new aggregate graph (Simpson et al., 2012; Gleichgerrcht et al., 2015; Wang et al., 2015; Yeh et al., 2018; Aggarwal and Gupta, 2019; Hallquist and Hillary, 2019; Domhof et al., 2021). However, this approach does not account for intersubject variability and thus may lead to significant deviation from the ground truth of the population. To overcome this pitfall, we proposed and tested a practical yet robust alternative approach. Instead of “aggregate-then-measure” approach, we adopted a reverse approach, namely “measure-then-aggregate” approach. For this, we first extracted graph rankings of each individual separately, and then averaged the corresponding rankings of all subjects (Figure 2). Indeed, this approach resulted in significantly higher correlations with the individual rankings, thus demonstrating higher representative power for population-level connectomics studies. This approach can be used by other researchers in the field of network neuroscience as an alternative or complementary to more sophisticated or computationally demanding statistical approaches existing in the literature (Aggarwal and Gupta, 2019).

Second, we investigated how the classical graph metrics are intercorrelated with one another when the structural connectivity of the human brain is concerned. It is no surprise that node degree has the highest correlation index with all five fundamental network measures (i.e., degree, strength, efficiency, coreness, betweenness) tested in this study. This finding echoes what is already known in neuroscience literature. The degree, which accounts for the total number of connections a node makes (Meskaldji et al., 2013), is one of the most common measures of centrality or hubness (Rubinov and Sporns, 2010). It is intuitive that high-degree nodes are interacting, structurally and/or functionally, with many other nodes in the network and thus play a crucial role in the system’s dynamics (Rubinov and Sporns, 2010; Bullmore and Bassett, 2011). However, there are other important characteristics of the network that may not be reflected by the degree itself and require the inclusion of other measures. Thus, we decided to average all five fundamental measures to obtain an overall “hubness” measure. By doing this, we aimed to find with higher accuracy (or probability) the hub regions of the brain that rank high in the order most consistently across all cortical regions and across all subjects of a given population. It appears that this overall “hubness” measure is indeed highly correlated (r_s_ > 0.9) with all graph measures tested. Although we are aware of the fact that such averaged metrics may mask more subtle or focal effects occurring in specific subsets of nodes as pointed out in the previous literature (Fornito et al., 2013), we think that it is still relevant for computational simplification and neuroscientific interpretation (Fornito et al., 2013).

Third, we sought to identify the minimum number of subjects required for accurate representation of population to account for interindividual variability in the studies of human connectome networks. Taking the HCP1065 dataset as the ground truth (i.e., population), we calculated correlations of randomly selected samples with the population average. We showed that a sample size of 100 subjects may be sufficient to represent the population with a margin of error as low as 1%. We think that this information might be useful for the future studies in determining the sample size and optimizing resources for data acquisition and analysis. The number of subjects used in similar studies in the connectome literature spans anywhere from a few to several hundred subjects (Farahani et al., 2019). In light of the present study, we think that some of these studies with smaller sample sizes should be cautiously interpreted or need to be supported with larger studies (Hagmann et al., 2008).

After the methodological optimization, we present our findings with regard to network properties of human structural connectome using this ranking-based approach. As clearly seen from the heatmaps, brain regions with high probability of hubness function are distributed over the entire cerebral cortex, but preferentially located in the medial surfaces of the hemispheres. On the other hand, lateral hemispheric surface has also important anatomical hub regions such as pars opercularis of inferior frontal gyrus, insula, and temporo-occipital area. These findings derived from a large dataset of subjects are mostly in line with the previous literature (Hagmann et al., 2008; Iturria-Medina et al., 2008; Bullmore and Sporns, 2009; van den Heuvel and Sporns, 2013). In addition, the present study revealed some previously underrecognized areas as hub regions such as cuneus and pars opercularis. Further in-depth graph theory applications could better delineate the importance of these regions within global and local networks. The fifth finding of the study was the striking asymmetry between two hemispheres. There are previous studies focused mostly on structural asymmetry of certain regions (Barrick et al., 2005; Otsuka et al., 2008; Dubois et al., 2009). Also, a limited number of studies have tried to explain hemispheric asymmetry using the connectome network (Shu et al., 2015). Although some homologous areas seem to share hubness function in both hemispheres, there are several parcels that rank considerably different between two hemispheres. This finding is in contrast with the previous literature that emphasizes hemispheric symmetry (Li et al., 2013). One might think that this finding could be due to a methodological artifact. To test this potential problem, we checked the MNI space coordinates of two parcellation atlases (AAL2 and HCP-MMP). Indeed, the sagittal midline crosses medial gyri more on the left hemisphere than the right when AAL2 atlas was used. But, when the HCP-MMP atlas was chosen, which was created as a comprehensive parcellation scheme based on actual HCP datasets, this midline crossing was much less evident. Therefore, we thought that using HCP-MMP atlas and having the entire set of subjects from the HCP-1065 dataset would overcome this technical problem. Yakovlevian torque could also be another explanation for this hemispheric asymmetry (Toga and Thompson, 2003). Nevertheless, if this finding is not just a result of a technical or methodological artifact; then it is worth more attention in the future brain connectivity studies. These ranking discrepancies of homologous brain regions in terms of graph measures could potentially point to an intrinsic distinct wiring, and could explain differential functional specialization of two hemispheres (i.e., hemispheric dominance).

Finally, we found considerable intraparcel heterogeneity similar to the hemispheric asymmetry. Even with the HCP-MMP atlas, which is considered as one of the most robust and comprehensive of all available atlases, some parcels have a high level of heterogeneity within their boundaries. Since the HCP-MMP atlas has parcels with diverse voxel sizes (30x difference between the smallest and the largest), we created subparcels within each of 180 parcels to obtain nearly 1,600 subparcels with similar sizes. This granularity scale was chosen to optimize spatial resolution and computational workload. Previous studies showed that parcellation technique and scale influence the network topology in some properties such as small-worldness (Zalesky et al., 2010). Romero-Garcia et al. (2012) found that statistical power of small-world properties decreases with highly grained scales, and cortical scale of around 600 regions represents the best trade-off between small-worldness and resolution of the cortical scale. Moreover, the spatial location of highly connected brain hubs (Bullmore and Bassett, 2011) and the relation between nodal characteristics and region size (Wang et al., 2009) were shown to be dependent on the atlas used (de Reus and van den Heuvel, 2013). Our findings indicate that intraparcel regional connectivity differences deserve more attention and possibly account for differential functional specialization of subregions within the same cortical parcels. As our results indicate significant intraparcel heterogeneities within structural connectome network, they question the generalizability of even the most robust parcellation atlases at population level (Eickhoff et al., 2018; Salehi et al., 2020). Also our intraparcel heterogeneity finding will likely give rise to new questions on the redefinition of eloquent brain regions.

Besides its several strengths such as the use of a large-scale, validated dataset, rigorous methodological optimization and new computational approach to study connectomics, our study has certain limitations, too. First of all, this study is solely based on structural connectivity data and does not account for functional connectivity. Second, this study utilized a deterministic tractography tool (DSI studio), however, it may be worth conducting similar analyses using different tractography algorithms (such as MRtrix3, which is uses probabilistic fiber tracking method) and different formulas to define the edge weights (such as SIFT2 in MRtrix3) and compare the results with the ones obtained in this study. Third, this study included only cortico-cortical connections but not cortico-subcortical ones. The parcellation atlas used here (HCP-MMP) as well as the intention for direct comparison with previous studies precluded the incorporation of subcortical structures, which could provide more accurate and complete picture of the structural connectivity network of the brain. Fourth, we have taken into account only five node-based graph metrics but not other global or local network properties. Fifth, group-level ranking-based analysis takes into account averages of relative rankings of graph measures but not absolute values, which may either mask or exaggerate actual connectivity patterns depending on individual differences. Sixth, even though it seems a consistent finding supported by objective data, there is still a chance that hemispheric asymmetry found in our study could be a result of artifact or technical error that was not appreciated in the context of experimental design of the present study. Further studies are needed to overcome the abovementioned limitations, validate our findings and reveal underlying differential connectivity patterns within two hemispheres. It is likely that there are important differences in fiber densities and trajectories of white matter tracts that may underlie this hemispheric asymmetry and intraregional heterogeneities. On the other hand, this study presents hints as to how the perspective of optimization theory can offer answers to existing network neuroscience questions. Thus, the use of data science and optimization applications can open new research avenues in network neuroscience.

## Conclusion

Advances in imaging and computational technologies have led to the explosion of connectomics data in the field of neuroscience. Despite numerous data analytical tools and approaches used in the field, there is still need for novel methodological approaches to better analyze and interpret big data of human brain connectomics. In this study, we have introduced and tested a novel mathematical approach to analyze network characteristics of human connectome on population level with a different perspective. First, we provided experimental evidence for the robustness, limits and applicability of this new population-averaging technique based on the hubness ranking of different nodes across the subjects of a given population. Second, we presented the novel findings and insights on the graph-based analysis of large HCP dataset using this new technique, which revealed striking hemispheric asymmetries and intraparcel heterogeneities in the structural connectivity of the human brain. Further investigations are warranted to confirm these findings and elaborate their implications for neuroscience.

## Data Availability Statement

The original contributions presented in the study are included in the article/supplementary material, further inquiries can be directed to the corresponding author/s.

## Ethics Statement

The studies involving human participants were reviewed and approved by Human Connectome Project (HCP). The patients/participants provided their written informed consent to participate in this study.

## Author Contributions

SH and TK contributed to the conception and design of the study. SB organized the database. TK performed the statistical analysis. SH, SB, and TK wrote the first draft of the manuscript. II, PC, EC, F-CY, and KO critically revised and wrote the sections of the manuscript. All authors contributed to manuscript revision, read, and approved the submitted version.

## Conflict of Interest

The authors declare that the research was conducted in the absence of any commercial or financial relationships that could be construed as a potential conflict of interest.

## Publisher’s Note

All claims expressed in this article are solely those of the authors and do not necessarily represent those of their affiliated organizations, or those of the publisher, the editors and the reviewers. Any product that may be evaluated in this article, or claim that may be made by its manufacturer, is not guaranteed or endorsed by the publisher.
